# Applying the Surgical Checklist Approach for Psychiatric Trainees to a Busy, Time Sensitive Psychiatric Crisis Environment

**DOI:** 10.1192/j.eurpsy.2023.304

**Published:** 2023-07-19

**Authors:** L. Celentano, A. Sadeghpour, D. Durand

**Affiliations:** University of Miami/ Jackson Memorial Hospital, Miami, United States

## Abstract

**Introduction:**

The Jackson Hospital Crisis (Psychiatric ED) Center serves the entirety of Miami-Dade County in Florida and is considered one of the top three busiest and per capita of clinical staff likely the busiest nationally across the United States. Simultaneously, the management of mental health emergencies has ascended near the top of international priorities driven by multiple trends (e.g., growing social isolation associated with the pandemic, de-stigmatization). The crisis environment presents a uniquely difficult challenge in applying high quality medical care: frequent suicidal gestures, impulsive often intoxicated or psychotic patients, unprecedented demand, diminishing staff capacity, and requirements to consistently apply specific medical and psychiatric protocols. These challenges are specifically difficult for new trainees who are not yet accustoms to the high acuity situations found in psychiatry. Moreover, academic, institutional, and governmental policies change on a monthly, or even weekly basis, often with the objective of improving care. Nevertheless, such constant shifts in workflow result in opportunities for errors that impact patient trajectories.

**Objectives:**

As such we endeavored to apply an evidence-based approach to improving the likelihood of success. One such vetted approach, the use of checklists, benefits from 10+ years of peer-reviewed research in “operational” medical specialties (e.g., surgery, ED, ICU) where work is segmented around interventions or time-shifts which share commonalities with the Psychiatric ED model. Yet the difficulty in implementing such models is typically not in the “hard” aspects of defining the checklist, but in the “soft” implementation dimensions of syndication, distribution, and enablement in the context of the individual unique settings.

**Methods:**

We developed a checklist that will be implemented on the Jackson Crisis emergency department, with the aim of having first year trainees complete the check list on each individual patient. Surveys were administered before implementation of checklist and will be distributed on a quarterly basis to trainees to evaluate for changes in resident confidence and clinical care.

**Results:**

Over six months, we launched this initiative with drivers of adoption focused on accelerating and easing new resident training, safety incident targeting, and in the balancing of “push” top/down and “pull” bottom/up dynamics. Initial learnings include rapidly improving resident satisfaction as well as various other impacts currently being observed throughout the institution.

**Conclusions:**

Overall reception to the checklist has been positive from attendings and new trainees alike, further evidence will be analyzed and presented as the checklist is utilized throughout the psychiatric ED.

**Disclosure of Interest:**

None Declared

